# What Is the Optimal Time on a Low-Calorie Diet Prior to Laparoscopic Anti-reflux Surgery? A Prospective Case-Controlled Study

**DOI:** 10.1007/s11605-022-05438-2

**Published:** 2022-08-25

**Authors:** Jessie Childs, Louise A. Mudge, Adrian Esterman, Sarah K. Thompson

**Affiliations:** 1 Allied Health and Human Performance, University of South Australia, Adelaide, Australia; 2 Adelaide Gastrointestinal Specialists, North Adelaide, South Australia Australia; 3 Clinical and Health Sciences, University of South Australia, Adelaide, Australia; 4 College of Medicine & Public Health, Flinders University, Rm 5E221.3, Flinders Medical Centre, Bedford Park, SA 5042 Australia

**Keywords:** Low-calorie diet (LCD), Very low-calorie diet (VLCD), Laparoscopic anti-reflux surgery, Bedside ultrasonography, Body composition analysis, Magnetic resonance imaging (MRI), Liver volume

## Abstract

**Introduction:**

A very low-calorie diet (VLCD) or low-calorie diet (LCD) is often used prior to laparoscopic surgery to optimize access to the hiatus. Much debate exists in the literature regarding the required duration for a VLCD or LCD, and how to evaluate the presence of a fatty liver. The aim of our study was to determine the optimal amount of time on an LCD to achieve maximal liver volume reduction, and to assess the accuracy of the InBody 230® vs. bedside ultrasonography vs. magnetic resonance imaging (MRI) in the measurement of liver volume.

**Methods:**

Seventeen consecutive patients undergoing laparoscopic anti-reflux surgery were recruited into the study. Each patient underwent body composition analysis with the InBody® 230, liver ultrasound, and liver MRI. Patients then began an LCD with a weekly ultrasound assessment until the day before surgery when they underwent repeat body composition analysis, liver ultrasound, and MRI.

**Results:**

The mean age was 54 years (range 21, 74). Maximal liver volume loss was noted within 3 weeks for 88% of participants, with 47% achieving their maximal liver volume reduction after the first week of an LCD. The mean reduction in liver volume was 16%, 18.6%, and 19% for MRI, ultrasound, and body composition analysis, respectively.

**Conclusion:**

Close to 90% of patients require 3 weeks or less on an LCD to achieve maximal liver volume loss prior to laparoscopic anti-reflux surgery. Body composition analysis and bedside ultrasonography were both as accurate as the gold standard MRI in the assessment of liver volume.

## Introduction

Obesity is the main risk factor for the development of non-alcoholic fatty liver disease. A large fatty liver may obscure the operative field during laparoscopic surgery in which access to the hiatus is critical (i.e., laparoscopic anti-reflux surgery, hiatus hernia repair, and bariatric surgery).^1^ In some instances, this may simply extend operating time; however, a large fatty liver may result in injury to the left liver lobe during retraction, and this in turn may require conversion to open surgery and/or cancellation of the surgery.

Some surgeons advocate a very low-calorie diet (VLCD; 450–800 kcal/day) or low-calorie diet (LCD; 800–1500 kcal/day) in the preoperative period to “shrink” the liver. A study examining liver volume after treatment with an Optifast® VLCD for 6 weeks with magnetic resonance imaging (MRI) found a 14.7% reduction in mean liver volume.^2^ The surgeons in this study felt that operability was improved due to better visualization of the gastro-esophageal junction, and easier retraction of the liver. A second multicenter, randomized, single-blind study found a significant reduction in postoperative complications in patients who received a 14-day VLCD prior to laparoscopic gastric bypass.^3^ However, there remains much controversy regarding the utility of a VLCD or LCD prior to laparoscopic non-bariatric upper gastrointestinal surgery, and little consensus on the optimal length of time required on such a diet to achieve maximal liver volume reduction.

Prior to surgery, estimation of a patient’s liver volume may be attempted by clinical examination using the palpation method; however, multiple studies have shown this technique to be inaccurate and potentially misleading.^4,5^ Whilst computed tomography (CT) and magnetic resonance imaging (MRI) remain the gold standard for measuring liver volume, they are expensive, not readily accessible, and carry various contraindications such as radiation exposure (CT) and claustrophobia (MRI).^6–8^ In contrast, ultrasound is non-invasive, non-radiating, fast and inexpensive. Child’s equation is a reliable and validated technique to calculate liver volume using three linear ultrasound measurements.^9–12^

The aim of our study was to determine the optimal amount of time on an LCD prior to laparoscopic anti-reflux surgery to achieve maximal liver volume reduction. We also wanted to use Child’s equation to measure change in liver volume following an LCD, and to assess its accuracy against MRI. If shown to be accurate, bedside ultrasonography could be used to confirm adequate liver shrinkage prior to surgery which in turn could lead to improved surgical outcomes.

## Methods

### Patient Selection and Study Design

Participants scheduled for laparoscopic anti-reflux surgery were recruited between 2018 and 2021. Participants who spoke English; were competent to give consent; were over 18 years old; and had a body mass index (BMI) of > 22 km/m^2^ were included. Those who had had a prior liver resection or were unable to undergo MRI were excluded. This study was conducted in accordance with the ethical standards of our institution (#0,000,036,306) and with the 1964 Helsinki declaration and its later amendments or comparable ethical standards.

Informed consent was obtained from all individual participants included in the study. An experienced dietitian assessed each patient and determined the optimal time on a preoperative LCD. At the outset of the study, each patient underwent body composition analysis with the InBody® 230, liver ultrasound, and liver MRI. Patients then underwent a weekly ultrasound assessment until the day before surgery where they underwent repeat body composition analysis, and liver ultrasound and MRI.

### Body Composition Analysis

One experienced dietician performed body composition analysis for all participants, at the time points specified above, using an InBody® 230 portable body composition analyzer (InBody Co., Ltd, Seoul, Korea). Body composition was evaluated with bioelectrical impedance analysis using two different frequencies (20 kHz and 100 kHz) of five body segments (right and left arms, trunk, right and left legs) with an 8-point tactile electrode system. Age, height, and gender were entered into the analyzer, and then weight, body fat mass, segmental fat mass, percent body fat, skeletal muscle mass, segmental lean mass, and total body water were all evaluated. Basal metabolic rate and recommended daily calorie intake was then predicted. The duration of the above measurements was approximately 30 s.

### Low-Calorie Diet

Following body composition analysis, all participants were instructed to consume three VLCD products per day (Optifast® VLCD, Optislim® VLCD, or Proslim Rapid VLCD), a minimum of two liters of low energy fluids, a minimum of two to three cups of low starch vegetables, and one teaspoon of oil. If a participant’s predicted skeletal muscle mass was on the lower side of normal, he/she was also instructed to include an additional entrée meal and, in individual cases, an additional snack, to optimize compliance and stem muscle mass wasting. The additional entrée meal generally consisted of 50–200 g of raw weight lean protein (e.g., lean red meat, chicken breast, fish or eggs) and, in individual cases, 20–40 g of carbohydrate. When a participant’s weight loss consistently fell below 1 kg per week, the dietician adjusted the LCD, either eliminating the additional prescribed protein and/or carbohydrate. The calorie and protein content of the LCD prescribed to each participant at baseline is included in Table 1. Participants were encouraged to include strength and resistance training and/or walking in their daily routine to stem muscle mass wasting. All participants weighed on their first day on an LCD and repeated this weekly. This information was reported to the dietitian via text messaging.

**Table 1:** Participant data showing number of weeks on a low-calorie diet (LCD) and total body fat lost

Participant number	Sex	Age	Pre BMI	Number of weeks on LCD	Total calories/day (kcal)	Total protein/day (g)	Post BMI	Total body fat lost (kg)
1	M	58	27.6	4	950	85	24.0	9.4
2	M	74	27.4	4	950	85	25.8	5.1
3	M	53	38.3	8	950	85	34.9	12.4
4	F	67	31.9	6	950	85	29.6	7.1
5	M	70	28.3	3	985	92	27.3	3.6
6	F	54	23.8	2	950	85	22.7	1.7
7	M	51	24.6	2	1205	101	23.8	5.7
8	M	45	29.6	3	1105	108	27.8	4.7
9	F	61	30.2	3	950	85	28.4	2.9
10	M	66	25.5	3	1105	108	24.0	5.8
11	M	27	29.8	2	1355	132	28.2	0.1
12	F	41	29.8	3	950	85	28.2	3.0
13	F	72	34.6	6	950	85	32.2	6.2
14	M	21	26.4	2	1305	122	24.4	3.9
15	M	63	27.3	4	1105	108	25.1	4.8
16	F	66	34.6	10	950	85	29.8	8.7
17	M	39	29.2	2	1005	97	27.5	2.1

### Liver Ultrasound

**Fig. 1:**
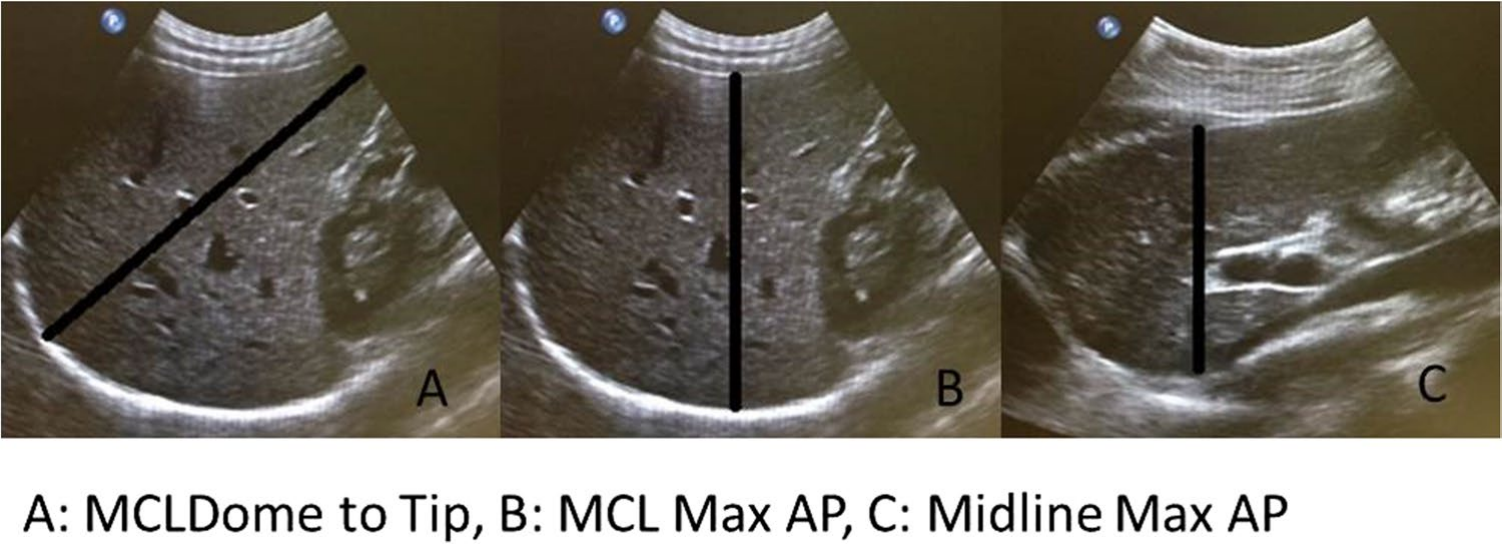
Ultrasound liver measurements where **A** = mid-clavicular line, dome to the tip of the liver; **B** = mid-clavicular line, anterior to posterior at liver’s maximum dimension; **C** = midline, anterior to posterior of the left lobe of liver

Two qualified and experienced sonographers performed all liver ultrasounds on the study participants. A Phillips IU22 ultrasound machine (Phillips Healthcare, Bothell, WA, USA) and a 5-–1-MHz curved array transducer measured liver volume according to the published protocol ^10^ (Fig. 1). The three linear measurements (A, B, C) were used to calculate liver volume (cm^3^) using the equation: 343.71 + [0.84 × ABC].

### Liver MRI

MRIs were performed on a Philips Ingenia 1.5 T MRI machine (Philips Healthcare, Bothell, WA, USA) with a 1.5-Tesla magnet. The MRI extended from just above the liver to just below the liver using a Axial e-THRIVE (T1 FEE 3D single shot) sequence with a 14-s breath hold. The MRI duration was approximately 10 min. Liver volume was calculated using the semi-automated Philips liver segmentation and analysis package (Eindhoven, The Netherlands) which has been shown to be a reliable method.^13^

### Statistical Analysis

In an equivalence test of means using two one-sided tests on data from a paired design, a sample size of 17 achieves 80% power at a 5% significance level when the true difference between the means is 0, the standard deviation of the paired differences is 4 cc, and the equivalence limits are − 3 cc and 3 cc. A paired-samples t-test was performed to compare the % liver volume loss measured by ultrasound comparable to the % volume loss measured by MRI. A similar analysis was performed to assess the difference between % liver volume reduction and % body fat mass reduction.

## Results

Seventeen participants were recruited, 11 males and 6 females. The mean age (SD) was 54 (15.5) years (range 21, 74). Mean height (SD) was 173 cm (11.1 cm) (range 155, 193). The number of weeks on an LCD ranged from 2 to 10 weeks with the mean (SD) length of time at 3.9 (2.3) weeks. The mean (SD) weight of the participants prior to an LCD was 87.6 (11.7) kg with a mean (SD) BMI of 29.3 (3.9). The mean (SD) weight of the participants the day prior to surgery was 81.4 (10.8) kg, with a mean (SD) BMI of 27.3 (3.2) (Table 1).

The mean (SD) reduction in MRI estimates of liver volume was 16 (10) % whilst the mean (SD) reduction in ultrasound liver volume was 18 (9) %. The mean (SD) reduction in body fat was 19 (11) % (Table 2). The volume reduction in cm^3^ as measured by both ultrasound and MRI can also be seen in Table 2.

**Table 2:** Liver volume reduction and body fat reduction whilst on a low-calorie diet (LCD). All modalities showed similar reductions

	Liver volume (cc) Ultrasound		Liver volume (cc) MRI		Body fat (units)	
	Mean	SD	Mean	SD	Mean	SD
At first measurement	1617.77	346.81	1776.65	387.47	27.03	11.08
Prior to surgery	1294.61	188.12	1459.11	229.43	21.9	9.45
% change	18.6	9.1	16	10	19	11

Comparison of the % liver volume loss measured by ultrasound to the % liver volume loss measured by MRI was not statistically significant (*P* = 0.7). A Cohen’s D of 0.21 showed the difference was relatively small. Comparison of the % volume loss measured by ultrasound to the % body fat loss measured using InBody scales was also not statistically significant (*P* = 0.8). A Cohen’s D of 0.08 showed that the % body fat loss measured by InBody scales was a comparative measure of % liver volume loss measured by ultrasound. Likewise, % liver volume reduction is a good estimator of % reduction in body fat.

All seventeen patients lost liver volume on a low-calorie diet. Notably, 47% of patients peaked their liver volume loss after the first week on an LCD. Twelve per cent achieved their maximal liver volume loss after the second week, and 29% after the third week. Only two patients, both with an initial BMI over 30, lost their maximal liver volume after 3 weeks on an LCD, both in the fifth week.

## Discussion

Our study was originally designed to answer two questions. First, to determine the optimal length of time on an LCD prior to laparoscopic anti-reflux surgery. And second, to confirm that bedside ultrasonography (using Child’s equation) is as accurate as the gold standard modality, magnetic resonance imaging (MRI), in the measurement of liver volume.

We found that 47% of participants lost most of their liver volume after only 1 week of an LCD. In fact, close to 90% of participants achieved maximal liver volume loss in the first 3 weeks of an LCD, suggesting that there is little value to enforcing a longer duration of a preoperative LCD. To our knowledge, only two other studies evaluated liver volume loss at shorter intervals, although no other study has evaluated it on a weekly basis. Gonzalez-Perez et al. evaluated liver volume with CT scan at 2-week intervals.^14^ Although not mentioned in their discussion, they found maximal liver volume reduction occurred within the first two weeks. Similarly, Colles et al. found that 80% of the size reduction in the liver occurred in the first 2 weeks as measured with serial CT scans.^15^ They found that visceral fat and overall weight decreased in a more uniform manner over the 12 weeks they kept patients on a VLCD.

Participants in our study lost on average 19% of their liver volume by following an LCD for a mean time of 3.9 weeks. This is in keeping with other studies which have reported a change in liver volume ranging from 5 to 20% for patients on a VLCD,^16^ and 12 to 27% for patients on an LCD.^17^ It is interesting to note that diets with lower caloric values did not result in greater liver volume loss than diets with higher caloric values.^16,17^

In the most recent systematic review,^17^ the authors found that studies with a shorter duration of an LCD (between 2 and 4 weeks) showed equivalent liver volume loss to studies with a longer duration of an LCD. The glaring exception to this is a study by Fris et al. in 2004 which reported only a 5.1% reduction in liver volume over a 2-week period on 456 kcal/day.^18^ Other authors have erroneously put this down to the short duration of the diet. However, our study refutes this theory, where we found 47% of participants lost their maximal liver volume after only one week on an LCD. We believe the low measurements may reflect the imaging method used in the study. Fris et al. used ultrasound to measure liver volume, however they did not use Child’s equation which involves the use of 3 imaging planes as described above (Fig. 1). Instead, Fris et al. used only two measurements of the left liver lobe: the maximum dorsal–ventral length anterior to the aorta, and the maximum cranio-caudal length. This is not a validated measure of liver volume.

As expected, we found that bedside ultrasonography (using Child’s equation) was as accurate as the gold standard modality, magnetic resonance imaging (MRI), in the measurement of liver volume. An unexpected finding was that the InBody® 230 body composition analyzer was also very accurate in the (indirect) measurement of liver volume, and no significant difference was noted between it and the other modalities. To our knowledge, this has not been shown before and highlights the utility of this device over a standard scale.

Several other points are worthy of discussion. A low-calorie diet (LCD) is an extremely effective method of decreasing liver volume prior to laparoscopic anti-reflux surgery. All seventeen participants showed excellent compliance on an LCD, and this may reflect both the ease with which an LCD is tolerated versus a VLCD, and the inclusion of a weekly “weigh in” by each patient to our dietician via text messaging.

It is also important to point out that ours is the first study to evaluate liver volume loss with an LCD in a largely non-bariatric population. All other studies have been conducted in bariatric surgery populations.^16,17^ Our study demonstrates unequivocally that an LCD is an effective method of reducing liver volume in a non-bariatric patient population. Since the conclusion of the study, we have implemented a 2-week LCD for patients with a BMI < 30 kg/m^2^. Those with a BMI over 30 kg/m^2^ remain on an LCD for a minimum of 3 weeks, guided by a dietician and the InBody® 230 body composition analyzer.

Our study had some limitations. We did not include any intra-operative complexity measures in our study design although all procedures were carried out by the senior author and no difficulties were encountered. These measures suffer from their subjective nature, and with greater laparoscopic expertise, minimal changes in liver volume will not affect operating times nor early complication rates in an appreciable manner. This is supported by Van Nieuwenhove et al.’s study, which found no reduction in perioperative complications with a VLCD.^3^ It is the authors’ view however that preoperative liver volume reduction increases the ease of the operation secondary to increased operative space/field of view. This may lead to better long-term outcomes due to more accurate identification of the fundus of the stomach for subsequent fashioning of the fundoplication.

## Summary

This prospective case-controlled study showed that close to 90% of patients achieved maximal liver volume reduction within three weeks on an LCD. In fact, 47% achieved this during the first week on an LCD. This was seen in a largely non-obese population as all patients were undergoing laparoscopic anti-reflux surgery. As well, we found that bedside ultrasonography (using Child’s equation) and the InBody® 230 body composition analysis device were both as accurate as the gold standard modality, magnetic resonance imaging (MRI), in the measurement of liver volume. These less expensive and portable methods could be used to confirm adequate liver shrinkage prior to surgery which in turn could lead to improved surgical outcomes.
